# A semi-supervised weighted SPCA- and convolution KAN-based model for drug response prediction

**DOI:** 10.3389/fgene.2025.1532651

**Published:** 2025-03-21

**Authors:** Rui Miao, Bing-Jie Zhong, Xin-Yue Mei, Xin Dong, Yang-Dong Ou, Yong Liang, Hao-Yang Yu, Ying Wang, Zi-Han Dong

**Affiliations:** ^1^ Basic Teaching Department, Zhuhai Campus of Zunyi Medical University, Zhu Hai, China; ^2^ Institute of Systems Engineering, Macau University of Science and Technology, Macau, China; ^3^ School of Biomedical Engineering, Guangdong Medical University, Dongguan, China; ^4^ Peng Cheng Laboratory, Shenzhen, China

**Keywords:** drug response prediction, feature extraction, sparse PCA, Kolmogorov–Arnold networks, data fusion

## Abstract

**Motivation:**

Predicting the response of cell lines to characteristic drugs based on multi-omics gene information has become the core problem of precision oncology. At present, drug response prediction using multi-omics gene data faces the following three main challenges: first, how to design a gene probe feature extraction model with biological interpretation and high performance; second, how to develop multi-omics weighting modules for reasonably fusing genetic data of different lengths and noise conditions; third, how to construct deep learning models that can handle small sample sizes while minimizing the risk of possible overfitting.

**Results:**

We propose an innovative drug response prediction model (NMDP). First, the NMDP model introduces an interpretable semi-supervised weighted SPCA module to solve the feature extraction problem in multi-omics gene data. Next, we construct a multi-omics data fusion framework based on sample similarity networks, bimodal tests, and variance information, which solves the data fusion problem and enables the NMDP model to focus on more relevant genomic data. Finally, we combine a one-dimensional convolution method and Kolmogorov–Arnold networks (KANs) to predict the drug response. We conduct five sets of real data experiments and compare NMDP against seven advanced drug response prediction methods. The results show that NMDP achieves the best performance, with sensitivity and specificity reaching 0.92 and 0.93, respectively—an improvement of 11%–57% compared to other models. Bio-enrichment experiments strongly support the biological interpretation of the NMDP model and its ability to identify potential targets for drug activity prediction.

## 1 Introduction

Precision oncology aims to leverage genomic information to identify patient groups with similar biological traits, enabling the delivery of the most suitable treatments (Dlamini et al., 2020; Garraway et al., 2013; Hodson, 2020; Prasad, 2016; Prasad et al., 2016). In clinical applications, this approach generally involves choosing targeted therapies based on the individual genomic profiles of patients (Ballester and Carmona, 2021). However, research reveals that only approximately 9% of patients experience effective outcomes from such targeted treatments, which greatly restricts the broad applicability of precision oncology (Barretina et al., 2012a; Rubio-Perez et al., 2015). Moreover, limited drug response prediction models for non-specific therapies mean that many patients miss out on the benefits of precision oncology and may even receive ineffective treatments. Fortunately, data from extensive pharmacogenomic screenings have shown that nearly all cancer cell lines and patient-derived xenografts (PDXs) respond to some form of targeted therapy or non-specific chemotherapy (Barretina et al., 2012b; Gao et al., 2015; Garnett et al., 2012). Thus, a primary challenge now is accurately aligning cancer patients with treatments that match their unique drug response profiles.

Currently, a significant research focus is predicting drug responses in cancer patients using single genomics data (Adam et al., 2020; Dong et al., 2015; Firoozbakht et al., 2022; Sheng et al., 2015). For instance, as demonstrated by Geeleher et al., a ridge regression model that utilizes gene expression data from the Genomics of Drug Sensitivity in Cancer (GDSC) database has shown effective application to clinical trial datasets for drugs including erlotinib, cisplatin, docetaxel, and bortezomib. The study also found that incorporating data from cancer cell lines other than breast cancer can improve the predictive performance of the docetaxel drug response model (Geeleher et al., 2014). Moreover, our preliminary research indicates that combining statistical methods based on individual genomic information from patients with machine learning techniques can construct highly performant drug response prediction models (Miao et al., 2020; Zheng et al., 2024; Sharma et al., 2024).

Recently, the increasing availability of multi-omics datasets for drug response has opened new avenues for machine learning models, enabling a deeper understanding of biological processes. Multi-omics data have shown notable success across various bioinformatics tasks, including survival prediction, cancer subtype classification, and target gene identification (Xu et al., 2024). As deep learning continues to progress rapidly, constructing predictive models that utilize multi-omics data through deep learning techniques becomes a primary research focus. Several multi-omics drug response models have been developed (Ballester et al., 2022; Baptista et al., 2021; Chen and Zhang, 2021; Zhou et al., 2024; Rashid, 2024; Baptista and Ferreira, 2023). For instance, Chiu et al. developed a deep learning model that utilizes autoencoders to combine diverse omics features for drug response prediction (Chiu et al., 2019). Similarly, Hossein et al. proposed a model that employs deep neural network fusion, combining hidden layer representations from different multi-omics networks to synthesize feature information effectively (Sharifi-Noghabi et al., 2019). Peng et al. proposed a two-space graph convolutional neural network (TSGCNN) that combines cell line and drug feature spaces to predict drug responses by leveraging both homogeneous and heterogeneous relationships (Peng et al., 2023). Similarly, Trac et al. proposed a GCN-based drug response prediction model for acute myeloid leukemia (AML), highlighting the versatility of graph-based neural networks in oncology research (Trac et al., 2023). Wang et al. proposed MOICVAE, a deep learning model that integrates multi-omics data using a variational autoencoder to improve drug sensitivity prediction (Wang et al., 2023). Meanwhile, Sharma et al. proposed DeepInsight-3D, architecture to fuse multi-omics data for anticancer drug response prediction, offering an advanced deep learning perspective for modeling complex interactions in diverse biological datasets (Sharma et al., 2023).

Currently, the multi-omics drug response prediction model faces three major challenges. First, genomic data typically involve small sample sizes, which increases the likelihood of overfitting in existing models (Deng et al., 2023). Developing an efficient and biologically interpretable feature selection method to select key genomic data is the first major challenge currently faced (Deng et al., 2023). Second, most genomic datasets for drug response prediction contain multiple independent genomic data types (Munquad et al., 2024). The data lengths and noise levels of these genomic datasets vary significantly, making the rational design of the multi-omics fusion method the second major challenge in constructing high-performance drug response medical models. Third, considering that drug response prediction is a complex biological problem and the dataset has only limited training samples, constructing a sufficiently high-performance prediction model based on a small sample of data remains the third major challenge.

**FIGURE 1:**
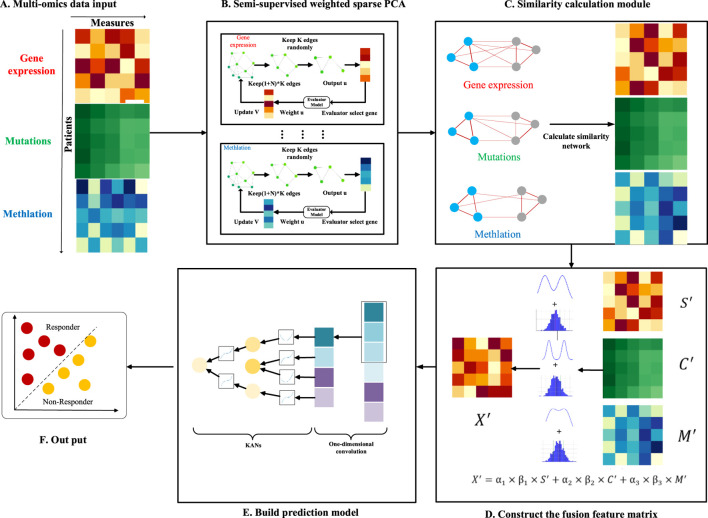
Overview of the NMDP model workflow. **(A)** Multi-omics data input. **(B)** Semi-supervised weighted sparse PCA. **(C)** Similarity calculation module. **(D)** Construct of the fusion feature matrix. **(E)** Prediction model. **(F)** Output.

In this study, we introduce an innovative model for predicting drug response (NMDP, Figure 1). The NMDP model is composed of four main modules. 1) Key genome selection module: we propose an interpretable, semi-supervised weighted sparse PCA to identify essential biological features. 2) Similarity network construction module: this module addresses the challenge of aligning data across different omics. 3) Data fusion module: we introduce a weighted similarity network fusion approach, incorporating the dip test method and variance information. 4) Drug response prediction module: we integrate one-dimensional convolutional neural networks (CNNs) and the Kolmogorov–Arnold network (KAN) method.

## 2 Materials and methods

### 2.1 Datasets

In this study, we use publicly available datasets to extract drug response and genomic data from cell lines. The first dataset is Genomics of Drug Sensitivity in Cancer (GDSC), which provides extensive data on drug response measurements. The second is the Gene Expression Omnibus (GEO) and Cell Model Passports, along with the European Bioinformatics Institute (EMBL-EBI) datasets. These two datasets provide the genomic data needed for this experiment.

It is important to note that the GDSC dataset comprises 624 unique drugs, 576,758 IC_50 values, and 978 cell lines. Genomic characteristics for each cell line include somatic copy number alterations (SCNAs) across 21,878 genes, RNA-Seq expression levels for 44,421 probes, and methylation levels for 365,860 CpG sites. For our study, we select 68 drugs: 14 FDA-approved targeted therapies, 49 drugs with known target genes not yet FDA-approved, and 5 nonspecific treatments (Supplementary Tables S1–S3).

### 2.2 Dataset of gene pathway data

The pathway data used in this study are sourced from the Pathway Commons database, which contains commonly used pathway datasets such as the Kyoto Encyclopedia of Genes and Genomes (KEGG) and Gene Ontology (GO).

### 2.3 Drug response data

In addition to the preprocessing already performed by the provider of this dataset, we also perform additional preprocessing. The following are the steps and criteria of our preprocessing: first, we remove samples with certain missing data, such as samples with a missing rate of more than 10%; second, we remove drugs with limited IC_50 test information, requiring the amount of IC_50 test data for each drug to be no less than 200 samples. Third, we use waterfall distribution to divide drug response data (Ding et al., 2018). Waterfall distribution is a method that sorts drugs based on their IC_50 values and uses a linear model to fit the data, which is used to determine whether a drug is effective. Specifically, the drugs are sorted according to the true IC_50 information. A linear model is then constructed to fit the distribution, and Pearson’s coefficient is used to evaluate the degree of fit of the model. If the fit is higher than 0.95, then the median is chosen as the cut-off point. If the fit is less than 0.95, a new monadic linear function is created, and the parameters of the function are determined by the smallest and largest points of IC_50. Finally, the point furthest away from the unary linear function in the IC_50 curve is calculated as the demarcation point. Ultimately, we classify divide drugs into two categories: responsive and non-responsive. In addition, to ensure that the data are balanced, we ensure that the response group constitutes at least 25% of the total data.

### 2.4 Methods

In this section, we provide a detailed overview of the architecture and algorithm flow of the NMDP model. This NMDP method transforms the sparse PCA model from a non-supervised to a semi-supervised approach, improving the ability of feature selection (key genome selection module). Second: Similarity network building module: in this module, we construct a sample similarity network based on the Spearman and Kendall correlation coefficients. Third: Data fusion module: we develop a data fusion algorithm based on the dip and variance tests. Fourth: Drug response prediction module: in this module, we propose a drug response prediction model based on one-dimensional convolution and KANs.

#### 2.4.1 Key genome selection module

##### 2.4.1.1 ESPCA method

Before introducing the NMDP model, we first define the sparse PCA (SPCA) and edge sparse PCA (ESPCA) models. Suppose we have an m×n feature matrix ${X\in R}^{m,n}$ , where n represents the number of samples and m represents the number of gene probes. The definition of SPCA is given by Formula 1:

$$\underset{{\left\|u\right\|}_{2}\leq 1}{maximize}u^{T}XX^{T}u,s.t.\;{\left\|u\right\|}_{0}\leq s.$$ (1)

Here, ${\left\|*\right\|}_{2}$ and ${\left\|*\right\|}_{0}$ represent $L_{2}$ and $L_{0}$ norms, respectively. u represents principal component (PC) loading, which has the dimension as the number of gene probes. s represents the retention number of gene probes. In most cases, the SVD method is used to solve Formula 1. Therefore, the formula can also be written as Formula 2:

$$\underset{{\left\|u\right\|}_{2}\leq 1,{\left\|v\right\|}_{2}\leq 1}{maximize}u^{T}Xv,s.t.{∥u∥}_{0}\leq s.$$ (2)

In this case, v represents the weight information corresponding to the sample, with dimensions matching the number of samples. ESPCA builds upon SPCA by incorporating improvements. Its main contribution is the integration of pathway structure information from the genome as *a priori* knowledge. Suppose that the known pathway structure information (edge set) is represented as $G=\left\{e_{1},\ldots ,e_{l}\right\}$ . At this point, the researcher introduces ${\left\|u\right\|}_{ES}$ regulon, which is represented as Formula 3:

$${\left\|u\right\|}_{ES}=\underset{\forall G^{\prime }\in G,support\left(u\right)\subseteq V\left(G^{\prime }\right)}{minimize}\left|G^{\prime }\right|.$$ (3)

Here, $G^{\prime }\in G$ and $V\left(G^{\prime }\right)$ represents the vertex set derived from the ${\left\|u\right\|}_{ES}$ regulon. Therefore, ESPCA can also be represented as Formula 4 (Min et al., 2018):

$$\underset{{\left\|u\right\|}_{2}\leq 1,{\left\|v\right\|}_{2}\leq 1}{maximize}u^{T}Xv,s.t.{∥u∥}_{ES}\leq s.$$ (4)

##### 2.4.1.2 Semi-supervised weighted edge sparse PCA

The existing SPCA and ESPCA methods are pure non-supervised methods; this method has a great advantage in data analysis with small samples and high dimensions. However, two primary issues arise: first, the method cannot utilize existing grouping information, which may reduce its effectiveness. Second, for the problem of drug response, the existing sparse PCA method selects the exact same key gene probe for all types of drugs; it obviously does not accord with the common sense of biology. In this study, we propose a novel semi-supervised weighted edge sparse PCA. This method mainly includes a weighted parameter t , which is calculated using a machine learning model. The parameter t leverages known grouping information on drug responses. Each time the model completes a cycle, we calculate t based on the currently selected key gene probes and weight u . Finally, we can select different key gene probes for each drug. The specific steps are shown in Formulas 5–12.

In general, the semi-supervised weighted edge sparse PCA method proposed in this paper can be expressed as Formula 5:

$$\underset{{\left\|u\right\|}_{2}\leq 1,{\left\|v\right\|}_{2}\leq 1}{maximize}u^{T}Xv,s.t.{∥u∥}_{NM}\leq k.$$ (5)

Here, ${\left\|u\right\|}_{NM}$ is a sparse regulon representing the edge group proposed by ESPCA and k is the regularization parameter. The regulon is given by Formula 6:

$${\left\|u\right\|}_{NM}=\underset{\forall G_{w}^{\prime }\in G_{w},support\left(u\right)\subseteq V\left(G_{w}^{\prime }\right)}{minimize}\left|G_{w}^{\prime }\right|.$$ (6)

Here, $G_{w}^{\prime }$ represents a subset of vertices selected from the edge set, with $\left|G_{w}^{\prime }\right|$ representing the count of vertices within this subset. Additionally, support $\left(u\right)$ represents the collection of non-zero elements in the sparse vector u . Then, we specifically explain how to calculate $G_{w}^{\prime }$ , supposing $e_{h}=\left(u_{i},u_{j}\right)\in G,u_{i},u_{j}\in R^{m}$ . At the beginning of the algorithm, v is randomly initialized. We use u=Xv to calculate the weight of u . Based on u , we use Formula 7 to calculate the edge weight $w_{h}$ corresponding to $e_{h}$ :

$$w_{h}=\sqrt{u_{i}^{2}+u_{j}^{2}}.$$ (7)

Finally, the edge weight can be represented as $G_{w}={\left\{w_{h}\right\}}_{1}^{l}$ . In this paper, we used a greedy principle based on the random sampling method, previously developed by our team, to sparsify u , as represented in Formula 8 (Miao et al., 2022):

$${\left[P_{G_{w}}\left(z,k\right)\right]}_{i}=\left\{\begin{array}{l} z_{i},if\;G_{w}\left(i\right)\cap sample\left(I,k\right)\neq \emptyset \\ 0,otherwise \end{array}\right..$$ (8)

Here, $P_{G}\left(z,k\right)$ represents the sparse projection, with ${\left[P_{G_{p}}\left(z,k\right)\right]}_{i}\left(i=1,\ldots ,m\right)$ . $I=supp\left({norm}_{G_{p}}^{DM}\left(e^{\prime }\right),\left(1+\omega \right)\times k\right)$ . $sample\left(I,k\right)$ represents the random selection of k elements from the set I . k denotes the number of non-zero elements selected in the sparsification process. If gene i is selected, then ${\left[P_{G}\left(z,k\right)\right]}_{i}=z_{i}$ ; otherwise, ${\left[P_{G}\left(z,k\right)\right]}_{i}=0$ .

In this case, we can obtain a sparse gene weight vector $\hat{u}=P_{G_{w}}\left(z,k\right)$ . Since existing sparse PCA models are non-supervised, identical input gene expression information results in the same $\hat{u}$ for each drug. In order to find a more suitable key gene set for different drugs, we design a linear evaluator based on machine learning, denoted as $\hat{y}=f_{\theta }\left(x\right)$ . For example, linear models or random forests can be used as evaluators. Here, θ represents the parameterized model, while the classification label corresponds to the drug response grouping information. Each time the sparse PCA model completes a cycle, we extract a new genome key expression matrix ${\hat{X}\in R}^{p,n}$ and $\hat{X}\in X$ based on $\hat{u}$ , where p represents the number of non-zero gene probes contained in $\hat{u}$ at that time. Next, $\hat{X}$ is input into $\hat{y}$ , as represented in Formula 9:

$$\theta^{*}=\underset{\theta }{\arg\;\min}L\left(\hat{X};\theta ,\omega \right).$$ (9)

Here, L represents the loss function. ω represents the optimizer of the model. $\theta^{*}$ represents the parameter after model training. Once training is complete, an importance score t is calculated for each gene probe associated with $\hat{X}$ . Finally, we obtain $t=\left\{t_{1},\ldots ,t_{p}\right\}$ . In order to ensure the stability in the weighting process, we perform a normalization step on t ,scaling the values to the range 0–2 . Finally, we update $\hat{u}$ based on t , as represented in Formula 10:

$$\hat{u}=\left\{t_{1}{\hat{u}}_{1},\ldots ,t_{m}{\hat{u}}_{m}\right\}.$$ (10)

In this case, if the gene probe corresponding to $t_{i}$ is not included in the set of t , then $t_{i}=0$ . We use Formulas 11, 12 to cross-update u :

$$u\leftarrow \frac{\hat{u}}{\left\|\hat{u}\right\|},$$ (11)

$$v\leftarrow \frac{\hat{v}}{\left\|v\right\|},where\;\hat{v}=X^{T}u.$$ (12)

Algorithm 1 Semi-supervised weighted edge SPCA.

1:u=Xv

$2:\text{for any weight of edge }e\text{ in }G_{w}\text{ do}$

$3:w_{h}^{\prime }=\sqrt{u_{i}^{2}+u_{j}^{2}}\;Generate\;a\;dynamic\;network.$

$4:{update\;G}_{w}^{\prime }=w_{h}^{\prime }$

5:end for

$6:\text{Let }{\text{norm}}_{G_{w}^{\prime }}^{NM}\left(e^{\prime }\right)={\left(∥e_{1}^{\prime }∥,\cdots ,∥e_{l}^{\prime }∥\right)}^{T}$

$7:I=\text{supp}\left({\text{norm}}_{C_{n}}^{NM}\left(e^{\prime }\right),\left(1+\omega \right)\times k\right)\text{ Extract }\left(1+\omega \right)\times k\text{ edges}$

$8:J_{k}=\text{sample}\left(I,k\right)$

9:if ω>0 then ω=ω−ρ

$10:V_{G_{w}^{\prime }}=V\left(G_{w}^{\prime }\right)$

$11:\text{for any gene }i\text{ in }V_{G_{w}^{\prime }}\text{ do}$

$12:{\hat{u}}_{i}=u_{i}$

13:end for

$14:\hat{X}=X\left[\hat{u}\right]$

$15:\text{Class}=\text{RandomForestClassifier }\left(\;\right)$

$16:\text{Class}.\text{ fit }\left(\text{ Class},Y\right)\;\backslash \#\text{Train and test the classifier}$

$17:t={\;\text{Class}.\;\text{feature}\;}_{\text{importances}\;}$

$18:\hat{u}=t*\hat{u}\;\#\text{ Weight }\hat{u}$

$19:\text{return }\hat{u}$

$20:u_{update}=\frac{\hat{u}}{∥\hat{u}∥}$

$21:v\leftarrow \frac{\hat{v}}{\left\|v\right\|},where\;\hat{v}=X^{T}u_{update}$

$22:loss={\left\|u-u_{update}\right\|}_{2},if\;loss<0.0001,end,then\;return\;step\;1$

#### 2.4.2 Similarity network building modules

For the same drug, we can get at least three different genomics data. The experiments in this paper mainly include gene expression data, copy volume data, and methylation data. Each omics performs sparse PCA operations independently. Finally, we can obtain three key feature matrices, namely, ${S\in R}^{k,n}$ , ${C\in R}^{p,n}$ , and ${M\in R}^{h,n}$ . k,p,h represents the number of key gene probes retained by each of the three omics.

Because of the inconsistency of the data lengths for each omics, data cannot be aligned. Therefore, we calculate a sample similarity subnet for each omics based on the concept of the sample similarity network. In this paper, we use two similarity measurement methods. We use the Spearman correlation coefficient to calculate the sample similarity subnet of gene expression and methylation omics, as represented in Formula 13:

$$\rho_{2}=\frac{\sum_{1}^{k}\left(x_{i}-\bar{x}\right)\left(y_{i}-\bar{y}\right)}{\sqrt{\sum_{1}^{k}{\left(x_{i}-\bar{x}\right)}^{2}\sum_{1}^{k}{\left(y_{i}-\bar{y}\right)}^{2}}}.$$ (13)

Here, for the x and y th sample, $x_{i},y_{i}$ represent the expression information on the i th gene expression in each sample, with k representing the total number of gene probes. The symbols $\bar{x},\bar{y}$ indicate the average gene expression levels for each sample.

The Kendall correlation coefficient is used for the copy number dataset, as provided in Formula 14:

$$Tau=\frac{C-D}{\frac{1}{2}k\left(k-2\right)}.$$ (14)

Here, the x and y th sample can be showed as a set of two elements containing p gene probe. C represents the number of consistent elements. D represents the number of inconsistent elements. k denotes the total number of gene probes in each sample.

#### 2.4.3 Data fusion module

After completing the construction of the sample similarity subnet, we can obtain three feature matrices, namely, $S^{\prime }{\in R}^{n,n}$ , ${C^{\prime }\in R}^{n,n}$ , and ${M^{\prime }\in R}^{n,n}$ . Then, we propose a subnet fusion algorithm based on the dip test and variance estimation using Formula 15:

$$X^{\prime }=\alpha_{1}\times \beta_{1}\times S^{\prime }+{\alpha_{2}\times \beta_{2}\times C}^{\prime }+{\alpha_{3}\times \beta_{3}\times M}^{\prime },$$ (15)

where $X^{\prime }$ represent the feature representation after fusion. α and β are defined as the amount of statistical information corresponding to the genomics data matrix. Theoretically, our goal is to retain as much of the feature matrix as possible, prioritizing genomics with higher statistical significance for drug response prediction. To achieve this, we use two statistical methods to assess the amount of information in the data. The first method is the single peak test, which aims to retain similarity matrices that exhibit more typical bimodal distributions. A bimodal distribution is a statistical concept that represents a dataset in more than two regions. In gene expression analysis, if the data show a bimodal part, it indicates a significant statistical difference within the sample. In this paper, we assess the bimodal property of data using the dip test method, originally proposed by Hartigan et al. (1985). We assume that $\rho \left(F,G\right)$ follows Formula 16 for any bounded functions F,G . Let μ be the class of unimodal distribution functions.

$$\rho \left(F,G\right)=\sup_{x}\left|F\left(x\right)-G\left(x\right)\right|.$$ (16)

We define μ as a typical unimodal distribution function and F as a dip distribution function. We can obtain Formulas 17, 18 as follows:

$$D\left(F\right)=\rho \left(F,\mu \right),$$ (17)

$$D\left(F_{1}\right)\leq D\left(F_{2}\right)+\rho \left(F_{1},F_{2}\right).$$ (18)

It is important to note that $D\left(F\right)=0$ for F∈μ , indicating that the dip quantifies deviation from unimodality. Assume that the result of the dip function is p , as shown in Equation 19:

$$\begin{array}{c} \;p>0.95:significant\;unimodality\\ \;p<0.05:significant\;bimodality \end{array}.$$ (19)

Another statistical method is the variance test. In addition to information about the probability distribution of the samples, our goal is to retain a matrix of sample similarity features that preserves as much discrete information as possible. The formula for the variance S information is provided in Formula 20:

$$S=\frac{\sum {\left(X-\bar{X}\right)}^{2}}{n-1},$$ (20)

where X is the variable, $\bar{X}$ is the sample mean, and n denotes the sample size. Suppose that the result of the variance of the i feature of the $i_{th}$ histology is $S_{ij}$ . Then, $\beta_{i}$ of $i_{th}$ histology can be expressed as Formula 21:

$$\beta_{i}=\frac{1}{n}\;\sum_{1}^{n}S_{ij}.$$ (21)

The computed $\beta =\left\{\beta_{1},\beta_{2},\beta_{3}\right\}$ accounts for the possibility that β having a large parameter. Therefore, we normalize β using Formulas 22, 23 as follows:

$$w_{i}=a+p\left(k_{i}-\mathrm{Min}\right),$$ (22)

$$p=\left(b-a\right)/\left(\mathrm{Max}-\mathrm{Min}\right).$$ (23)

Here, a and b are user-defined parameters, representing the normalized range of data. p represents a scaling factor used to normalize the raw data β to a user-defined range. Max and Min represent the maximum and minimum values of β , respectively.

#### 2.4.4 Drug response prediction module

**FIGURE 2:**
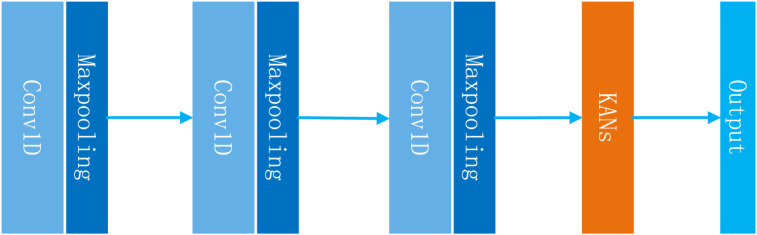
Structure of the deep learning model used in the NMDP model.

Finally, we obtain the feature matrix $X^{\prime }$ . Although the problem of the high dimensionality of data has been largely alleviated after genomic feature extraction and similarity network computation, researchers still need a powerful enough deep learning model to achieve high performance and avoid overfitting. However, considering the limitation of the number of samples, researchers still need a sufficiently powerful drug response prediction deep learning model to avoid model overfitting. In this study, we construct a deep learning model based on one-dimensional convolution and KANs to predict drug response (Figure 2). One-dimensional convolution can further localize the features of the samples and remove potential noise. Experimental results indicate that one-dimensional convolution significantly enhances the model’s prediction performance. KANs, proposed by Liu et al. (2024), aim to replace the traditional fully connected neural network layer. The network is based on the Kolmogorov–Arnold theorem, which states that any continuous function $f\left(x\right)$ in n-dimensional real space, where $x=\left(x_{1},\ldots ,x_{n}\right)$ can be represented as a combination of a single-variable continuous function h and a series of continuous bivariate functions $g_{i}$ and $g_{i,j}$ . Specifically, the theorem is expressed in Formula 24:

$$f\left(x_{1},\ldots ,x_{n}\right)=\sum_{q=0}^{2n}h\left(\sum_{i=1}^{n}g_{q,i}\left(x_{i}\right)\right).$$ (24)

The theorem shows that even a complex function in a high-dimensional space can be reconstructed using a series of lower-dimensional function operations. Specifically, a KAN layer with $n_{in}$ dimensional inputs and $n_{out}$ dimensional outputs can be defined as a matrix of one-dimensional functions, as represented in Formula 25:

$$KANs=\left\{\phi_{q,p}\right\},p=1,2,\ldots ,n_{in},q=1,2,\ldots ,n_{out},$$ (25)

where the function ϕ is defined as shown in Formula 26 and consists of a B-spline curve and a residual activation function $b\left(x\right)$ , all multiplied by a learnable parameter w . The function ϕ is defined as shown in Formula 26:

$$\phi =w_{1}\times spline\left(x\right)+w_{2}b\left(x\right).$$ (26)

The main advantage of KANs is that they can achieve results beyond fully connected neural networks while using fewer parameters. This is especially important for the drug response prediction problem. Due to the limitation of the sample size, it is unlikely that we can construct a deep learning model that contains a huge neural network. To summarize, the module can be expressed using Formulas 27, 28 as follows:

$$\overset{´}{X}=One-Dimensional\;Convolution\left(\;X^{\prime }\right),$$ (27)

$$out=KANs\left(\overset{´}{X}\right).$$ (28)

## 3 Results

The procedure in this article consists of six distinct steps. Initially, experiments were performed using 14 FDA-approved targeted therapy drugs already authorized for clinical use. In the second step, we broadened the model evaluation by testing it with 49 targeted therapy drugs not approved by the FDA. In the third step, we conducted experiments on five chemotherapeutic agents (non-targeted therapeutics) in order to verify that the NMDP model has good scalability. We used seven state-of-the-art AI models for comparison, namely, TSGCNN, MOICVAE, MOLI, netDx, netDx–elastic network, deep autoencoder, and netDx–SVR. Five evaluation indicators were used, namely, sensitivity, specificity, precision, accuracy, and F1 score. The details of comparison models are provided in Supplementary Materials.

In the fourth step, we selected the GDSC1 dataset for training and testing and the GDSC2 dataset for validation. We selected 14 FDA-approved drugs to perform and calculate the mean value. The consistency and reliability of the results were ensured by calculating the mean value.

The fifth step included conducting ablation experiments to determine the importance of each sub-module of the NMDP model. We randomly selected 10 drugs for analysis and averaged the results. All experiments were performed using the GDSC2 dataset (Supplementary Table S7). We conducted four independent experiments: the first experiment was conducted to remove the multi-omics weighting module; the second experiment, to remove the convolution module; the third experiment, to remove the sample similarity network; and the last experiment, to replace KANs with MLPs.

Ultimately, we used the Metascape platform to examine the biological pathways associated with the gene probes chosen by the NMDP model (Zhou et al., 2019). The details of the indicators are provided in Supplementary Materials.

### 3.1 FDA-approved targeted therapy drugs

**FIGURE 3:**
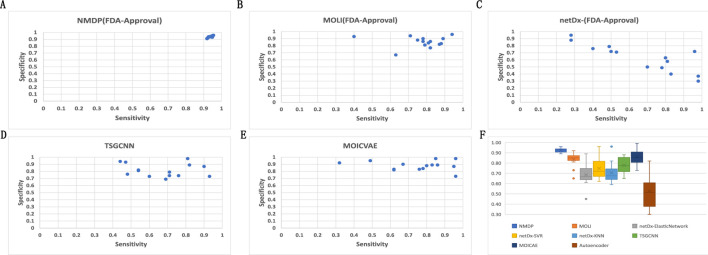
Results of 14 FDA-approved drugs for each model. **(A)** Sensitivity and specificity of the NMDP model; **(B)** sensitivity and specificity of the MOLI model; **(C)** sensitivity and specificity of the netDx model; **(D)** sensitivity and specificity of the TSGCNN model; **(E)** sensitivity and specificity of the MOICVAE model; and **(F)** accuracy of each model.

Based on the results presented in Figure 3 and Supplementary Table S4, experiments show that the NMDP model is much better than the advanced deep learning model. Notably, the NMDP model achieves an average sensitivity of 0.92 and a specificity of 0.93. Among the models for comparison, the MOICVAE model ranks highest, with a sensitivity of 0.77 and a specificity of 0.91. The deep autoencoder model, however, performs the lowest, with sensitivity and specificity values of 0.53 and 0.44, respectively.

Based on Figure 3C, it is evident that the deep autoencoder model exhibits overfitting across multiple drugs. Moreover, the NMDP model demonstrates minimal fluctuation across 14 drugs, indicating its superior stability (Figure 3F). Compared to other models, all except the MOLI model show relatively high levels of fluctuation, suggesting weaker stability in those models. The NMDP model achieves values of 0.93, 0.92, 0.92, and 0.92 for average accuracy and F1 score, outperforming the MOICVAE model by 10% in each metric (Table 1). When compared to the deep autoencoder model, it shows improvements of 31%, 48%, 52%, and 50%, respectively.

**TABLE 1:** Results of each model of the 14 drugs approved by the FDA.

		NMDP	MOLI	Deep autoencoder	netDx–KNN	netDx–ElasticNet	netDx–SVR	TSGCNN	MOICVAE
Accuracy	All	0.93	0.84	0.64	0.70	0.71	0.74	0.78	0.86
F1 score	Responsive	0.92	0.83	0.48	0.72	0.67	0.71	0.74	0.74
	Non-responsive	0.92	0.83	0.44	0.56	0.58	0.66	0.78	0.86
	All	0.92	0.83	0.46	0.64	0.63	0.68	0.77	0.80

**FIGURE 4:**
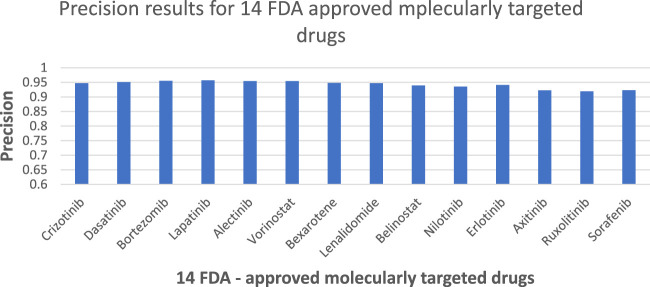
NMDP model precision results of 14 FDA-approved drugs.

According to Figure 4, the NMDP model demonstrated excellent performance in predictive accuracy. It is worth mentioning that out of these 14 drugs, the prediction accuracy for 13 of them exceeded 90%.

### 3.2 FDA non-approved targeted therapy drugs

**FIGURE 5:**
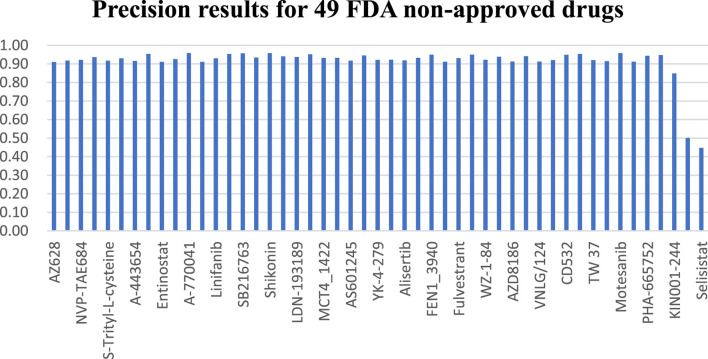
NMDP model precision results of five non-specific therapeutic drugs.

Across the 49 drugs not approved by the FDA, we observe similar outcomes. Experimental findings indicate that the NMDP model achieves average sensitivity and specificity values of 0.92 and 0.93, respectively, outperforming the comparison models by 11%–57% (Supplementary Figure S1; Supplementary Table S5). Supplementary Figure S1F illustrates the NMDP model’s high stability, with only 5 out of the 49 drugs showing precision below 0.9 (see Figure 5). In addition, the average precision of the NMDP method can reach 0.95. F1 score can reach 0.92, surpassing the MOLI model by 14%, 10%, and 13% (Supplementary Table S1). Among the seven models compared, MOLI achieves the highest performance. Nevertheless, its sensitivity, specificity, precision, and accuracy for response, non-response, and all samples are only 0.80, 0.88, and 0.86, respectively, with F1 scores of 0.83, 0.83, 0.83, 0.82, and 0.84.

### 3.3 Non-specific therapeutic drugs

**FIGURE 6:**
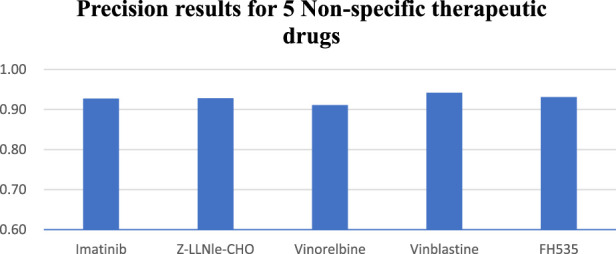
NMDP model precision results of five non-specific therapeutic drugs.

In the study of five non-specific therapeutic drugs, we achieve optimal outcomes in three experiments. Results indicate that the NMDP model achieves 0.93 for average sensitivity, surpassing the comparison models by 19%–42% (Supplementary Figure S2; Supplementary Table S6). Additionally, the NMDP model demonstrates a precision close to 0.95 across the five drugs (Figure 6). The model also shows outstanding performance in F1 score and accuracy, reaching 0.93 (Supplementary Table S2). Compared to the other models, the MOLI model achieves the best results, but its average F1 score and accuracy reach only 0.78. The experimental results show that the NMDP model has good expansibility.

### 3.4 External independent validation results

To evaluate the model’s performance and test its generalization ability, we design this external independent validation experiment. The experimental outcomes demonstrate that the NMDP model exhibits superior generalization capabilities. Specifically, the NMDP model achieves an overall prediction accuracy of 0.77 and precision, recall, F1 score, and accuracy of 0.73, 0.77, 0.74, and 0.77, respectively (Table 2). It is worth noting that a slight decrease in accuracy is observed on the validation set compared to the results on the test set. This may be due to the noise difference between the datasets. Overall, the NMDP model exhibits robustness and reliability, with the capability for widespread application.

**TABLE 2:** External independent validation results.

	Precision	Recall	F1-score
Responsive	0.72	0.74	0.73
Non-responsive	0.83	0.81	0.82
Accuracy			0.77
Macro average	0.73	0.77	0.74
Weighted average	0.8	0.78	0.79

### 3.5 Ablation experiment

The experimental results show that the model feature extraction effect is weakened by removing the multi-omics weighting module and the convolution module, but the convolution module has a greater impact on the model. The sample similarity network module has the greatest impact, further verifying the importance of similarity across samples. When KANs are replaced with MLPs, the performance of the model improves but still does not surpass that of the original model. This indicates that KANs have a unique advantage in capturing complex relationships, especially when dealing with multi-omics data. Taken together, the results of the ablation experiments fully indicate that the sample similarity network and convolution module are the key factors in improving the model performance. Among them, the sample similarity network module has the greatest impact, and we believe that the main reason is that, even after the feature filtering of the sparse PCA model, the three modules are still able to save more than 9,000 gene probes collectively, and the excessively high data dimensions make it easy for the model to fall into an overfitting state.

### 3.6 Enrichment analysis

**FIGURE 7:**
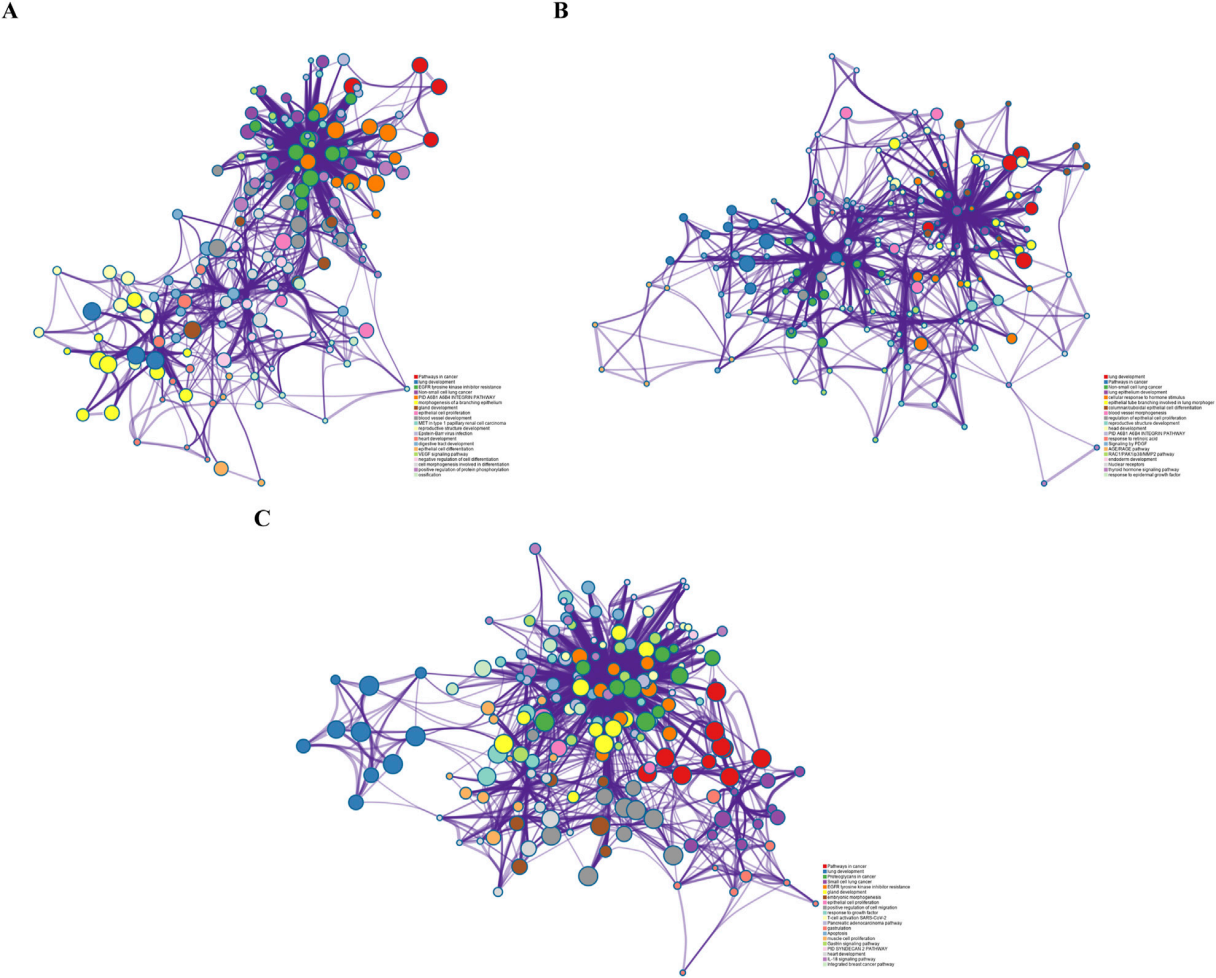
**(A)** Pathway results from the first PC of copy number; **(B)** pathway results from the first PC of methylation data; and **(C)** pathway results from the first PC of sequencing data.

To validate the biological interpretability of the NMDP model, we conduct bio-enrichment analysis using gene selection results for erlotinib across different omics types obtained from the first principal component (PC) in the NMDP model. Erlotinib is an FDA-approved non-small cell lung cancer drug, with EGFR as its primary target (Tsao et al., 2005; Zhou et al., 2021). The analysis yields promising results as the NMDP model successfully identifies lung cancer-related pathways and gene probes. For instance, in copy number omics, we discover pathways corresponding to genes like *EGFR*, *CDK6*, *RASSF5*, *BRAF*, and *CCND1*, which are associated with non-small cell lung cancer (Chen et al., 2018; Xue et al., 2019; Zhao et al., 2018) (Figure 7A). In methylation omics, we observe the developmental process pathway GO:0032502, involving genes such as *EGFR*, *FGFR2*, *GATA6*, *ASCL1*, *BMP4*, and *FOXA1*, indicating relevance to lung development (Bach et al., 2018; Ju et al., 2019; Murai et al., 2015) (Figure 7B). In Seq omics, we identify pathways related to the KEGG pathway, which include genes like *EGFR*, *MAPK1*, *KRAS*, *CCND1*, *HRAS*, *NRAS*, *PLCG1*, and *GRB2*, which show strong connections to non-small cell lung cancer (Betticher et al., 1996; Park et al., 2020; Pązik et al., 2021) (Figure 7C). Remarkably, EGFR, the target gene of erlotinib, is consistently identified across these three omics types.

Overall, the NMDP model demonstrates superior performance compared to other models across all metrics.

## 4 Discussion

With advancements in bioassay technology, a growing number of large-scale drug response datasets are being released, creating new possibilities for building drug response prediction models. In recent years, researchers have proposed a number of AI-based drug response prediction models (Chiu et al., 2020). However, drug response prediction data are mostly characterized by the typical features of multi-omics, small samples, high dimensionality, and high noise. Designing feature selection and multi-omics fusion methods based on regularization ideas becomes very important. In addition, considering the potential overfitting problem, it is difficult for researchers to build AI-based drug response prediction models with many parameters.

In light of the aforementioned issues, we propose the NMDP model, which integrates semi-supervised weighted SPCA, similarity networks, dip tests, and KANs. Unlike the traditional unsupervised sparse PCA model, the NMDP model proposes an independent evaluator that converts the sparse PCA model from a traditional unsupervised to a semi-supervised model. This improvement allows the NMDP model to use known dataset grouping information, ultimately allowing the model to stably select different potential target genes for different target drugs. The experimental results show that the NMDP model inherits the advantages of the sparse PCA model, such as good biological interpretability and strong denoising ability, further enhances the feature selection ability of the model in multi-omics gene data, and greatly strengthens the stability of the model in high-dimensional small-sample cases. Sample similarity networks further address the dimensionality challenge of the samples while helping the model perform multi-omics data alignment. We introduce a fusion algorithm that utilizes both dip test and variance data with weighted integration, which allows the model to focus on important histological information, thus improving prediction accuracy. Finally, we propose a one-dimensional convolution combined with KANs for drug response prediction modeling. The model achieves efficient prediction with a small number of parameters, thereby effectively avoiding the overfitting problem.

To enhance the validation of the model, we also conduct external validation experiments to assess the generalization capability of the model using independent datasets. The experimental findings indicate that the NMDP model performs consistently on different datasets, validating its robustness and reliability. In addition, we conduct ablation experiments to evaluate the contribution of each component to the model performance. The results of the ablation experiments show that removing the multi-omics weighting module and the convolution module significantly degrades the model’s performance, and in particular, the sample similarity network module plays the most crucial role in influencing the model’s effectiveness. This further emphasizes the importance of inter-sample similarity and the unique advantage of KANs in capturing complex relationships. Bioenrichment experiments fully validate the biointerpretability of the model, suggesting that the NMDP model could help researchers in drug development.

We also acknowledge some limitations to this study: our research is confined to predicting the response to a single drug, without considering the effects of combination drug therapies. Moreover, the weighted edge sparse PCA method has high time complexity, which leads to slower model computations. In future work, we plan to improve the model’s ability to predict responses to drug combinations and optimize its computational efficiency.

## Data Availability

The data presented in the study are deposited in the European Genome-phenome Archive (EGA) repository, accession numbers EGAD00001001039, EGAD00001004201, EGAD00010000644; and in the Gene Expression Omnibus (GEO) repository, accession number GSE68379.
