# Supplementary material for: A semi-supervised weighted SPCA- and convolution KAN-based model for drug response prediction
Source: Front Genet. 2025 Mar 21;16:1532651. doi: 10.3389/fgene.2025.1532651 (PMC11968432; doi:10.3389/fgene.2025.1532651)
Supplement: Supplementary file 3 [file Table7.DOCX]

Supplementary Materials

NMDP: A novel Multi-omics Drug response prediction model based on weighted edge sparse PCA and KANs

Rui Miao1*+, Bing-Jie Zhong1+, Xin-Yue Mei1, Xin Dong1, Yang-Dong Ou 1, Yong Liang3, Hao-Yang Yu1, Ying Wang1 and Zi-Han Dong1

1 Basic Teaching Department, Zhuhai Campus of Zunyi Medical University, Zhu Hai, China.

2 Institute of Systems Engineering, Macau University of Science and Technology, Avenida Wai Long, Taipa, Macau, China.

3 Peng Cheng Laboratory, Shenzhen, 518055, China

*** Corresponding Author:** miaorui.researh@gmail.com

**Supplementary Figure S1**

**Supplementary Figure S2**

**Supplementary Figure S3**

**Supplementary Table S1**

**Supplementary Table S2**

**1. Evaluation indicators**

## 1.1 Accuracy

Accuracy refers to the ratio of the number of correctly predicted samples to the total number of predicted samples. This is one of the most common classification indicators. However, this indicator cannot fully evaluate the model when the data is not balanced. Therefore, we also uses Precision, Recall and F1-score as comparison indicators.

## 1.2 Precision

Precision refers to the ratio of the number of correctly predicted positive samples to the number of all predicted positive samples.

## 1.3 Recall

Recall refers to the ratio of the number of correctly predicted positive samples to the total number of true positive samples.

## 1.4 F1-score

F1-score is equivalent to the harmonic average of precision and recall, and the result will refer to both indicators at the same time.

**2. Comparision model**

This section mainly introduces the comparison models used in the experiment including deep autoencoder, MOLI, netDX model, TSGCNN and MOICVAE.

In 2019, Chiu et al. proposed a multi-omics drug response prediction model based on a deep autoencoder. The model contains three deep neural networks (DNNs), i) a mutation encoder pre-trained using a large pan-cancer data set (The Cancer Genome Atlas; TCGA) to abstract core representations of high-dimension mutation data, ii) a pre-trained expression encoder, and iii) a drug response predictor network integrating the first two subnetworks. [1].

In addition to deep autoencoders, some researchers have also proposed an end-to-end multi-omics deep learning drug response prediction model. Hossein et al. proposed a multi-omics late integration model based on deep neural networks. MOLI takes somatic mutation, copy number aberration and gene expression data as input, and integrates them for drug response prediction. MOLI uses type-specific encoding sub-networks to learn features for each omics type, concatenates them into one representation and optimizes this representation via a combined cost function consisting of a triplet loss and a binary cross-entropy loss[2].

To compare whether the performance of the NMDP model is better than the existing models, we also selected a machine learning prediction model of the sample similarity network for comparison. We used netDx, a new supervised patient classification framework based on patient similarity networks[3]. In this article, we have also expanded the netDx model. We use the sample similarity network integration method of the netDx model for multi-omics fusion and use 3 machine learning methods to predict the drug response including: Elastic network, SVR,KNN.

In 2023, Peng et al. proposed a drug response prediction model based on a two-space graph convolutional neural network (TSGCNN). The model first constructs a cell line feature space and a drug feature space and performs graph convolution operations separately on these spaces to propagate similarity information among homogeneous nodes. Next, it builds a heterogeneous network using known relationships between cell lines and drugs and performs graph convolution operations on this network to gather features from different types of nodes. The final representations for cell lines and drugs are generated by combining their self-features, feature space representations, and heterogeneous space representations. A linear correlation coefficient decoder is then used to reconstruct the cell line-drug correlation matrix for drug response prediction [4].

In 2023, Wang et al. proposed a deep learning model called Multi-Omics Integrated Collective Variational Autoencoder (MOICVAE) to improve drug sensitivity prediction. This method first uses a multimodal autoencoder (MDA) to fuse transcriptomic and genomic data (including mRNA, CNV, and SNP) into a low-dimensional latent space. The fused omics feature vectors are then used to train a collective variational autoencoder (cVAE) for drug sensitivity prediction. MOICVAE demonstrated excellent performance on the GDSC and CCLE datasets, achieving high AUC scores, and also performed well on the TCGA dataset, showing strong predictive ability in drug sensitivity classification [5].

**3. Feature selection of NMDP**

In the real dataset experiments, we optimize the parameters involved in the entire experiment using a randomized grid search method to find a set of optimal parameters that balance model performance and complexity. This method allows the model to select the most appropriate number of features for each drug and each genomic data type, thereby improving prediction accuracy and biological relevance.

In general, we retain 1500-2000 RNA-seq probes features, 3000-4000 methylation probes features and 3000-4000 copy number probes features.

**Supplementary Figure S1** The result of each model in 49 FDA-non-approved drugs. A-E: Sensitivity and specificity of each model; F: Accuracy of each model. A-E:

**Supplementary Figure S2** The result of each model in 5 Non-specific therapeutic drugs. A-E: Sensitivity and specificity of each model; F: Accuracy of each model.

**Supplementary Table S1.** The result of each model of 49 Non-FDA-approved drugs

|  |  | NMDP | MOLI | Deep autoencoder | netDX | netDX-ElasticNet | netDX-SVR | TSGCNN | MOICVAE |
| --- | --- | --- | --- | --- | --- | --- | --- | --- | --- |
| Accuarcy | All | **0.91** | 0.82 | 0.65 | 0.65 | 0.68 | 0.71 | 0.75 | 0.83 |
| F1 score | Response | **0.91** | 0.78 | 0.42 | 0.65 | 0.57 | 0.71 | 0.67 | 0.74 |
|  | Non-Response | **0.92** | 0.83 | 0.54 | 0.54 | 0.66 | 0.65 | 0.76 | 0.84 |
|  | All | **0.92** | 0.80 | 0.46 | 0.64 | 0.61 | 0.68 | 0.74 | 0.79 |

**Supplementary Table S2.** The result of each model of 5 Non-specific therapeutic drugs

|  |  | NMDP | MOLI | Deep autoencoder | netDX | netDX-ElasticNet | netDX-SVR | TSGCNN | MOICVAE |
| --- | --- | --- | --- | --- | --- | --- | --- | --- | --- |
| Accuarcy | All | **0.93** | 0.78 | 0.57 | 0.66 | 0.70 | 0.74 | 0.76 | 0.81 |
| F1 score | Response | **0.93** | 0.64 | 0.30 | 0.45 | 0.27 | 0.37 | 0.48 | 0.48 |
|  | Non-Response | **0.93** | 0.82 | 0.76 | 0.70 | 0.83 | 0.84 | 0.85 | 0.91 |
|  | All | **0.93** | 0.78 | 0.47 | 0.58 | 0.55 | 0.61 | 0.66 | 0.70 |

**Reference**

1. Chiu, Y.-C.; Chen, H.-I.H.; Zhang, T.; Zhang, S.; Gorthi, A.; Wang, L.-J.; Huang, Y.; Chen, Y. Predicting drug response of tumors from integrated genomic profiles by deep neural networks. *BMC medical genomics* **2019**, *12*, 143-155.

2. Sharifi-Noghabi, H.; Zolotareva, O.; Collins, C.C.; Ester, M. MOLI: multi-omics late integration with deep neural networks for drug response prediction. *Bioinformatics* **2019**, *35*, i501-i509.

3. Pai, S.; Hui, S.; Isserlin, R.; Shah, M.A.; Kaka, H.; Bader, G.D. netDx: interpretable patient classification using integrated patient similarity networks. *Molecular systems biology* **2019**, *15*, e8497.

4. Peng, W.; Chen, T.; Liu, H.; Dai, W.; Yu, N.; Lan, W. Improving drug response prediction based on two-space graph convolution. *Computers in Biology and Medicine* **2023**, *158*, 106859.

5. Wang, C.; Zhang, M.; Zhao, J.; Li, B.; Xiao, X.; Zhang, Y. The prediction of drug sensitivity by multi-omics fusion reveals the heterogeneity of drug response in pan-cancer. *Computers in Biology and Medicine* **2023**, *163*, 107220.
